# The Good Physician; Being an Introductory to the Course of Lectures on Materia Medica and Therapeutics, in the Medical Department of Transylvania University, for the Session 1842-3

**Published:** 1842-12-24

**Authors:** 


*The Good Physician; being an Introductory to the Course of Lectures on Materia Medica and Therapeutics, in the Medical Department of Transylvania University, for the Session* 1842–3. By THOMAS D. MITCHELL, M. D., Professor, &c. &c. Lexington, Ky.: 1842. 8vo. pp. 18.

Dr. Mitchell sketches his *beau ideal* of the "good physician" in a sprightly and atractive manner, but we fear his standard is too high for successful imitation.

